# Supplementary material for: Tracking an untracked space debris after an inelastic collision using physics informed neural network
Source: Sci Rep. 2024 Feb 9;14:3350. doi: 10.1038/s41598-024-51897-9 (PMC10858253; doi:10.1038/s41598-024-51897-9)
Supplement: Supplementary file 1 — Supplementary Figures. [file 41598_2024_51897_MOESM1_ESM.pdf]

Appendix

Models trained using 4000 samples

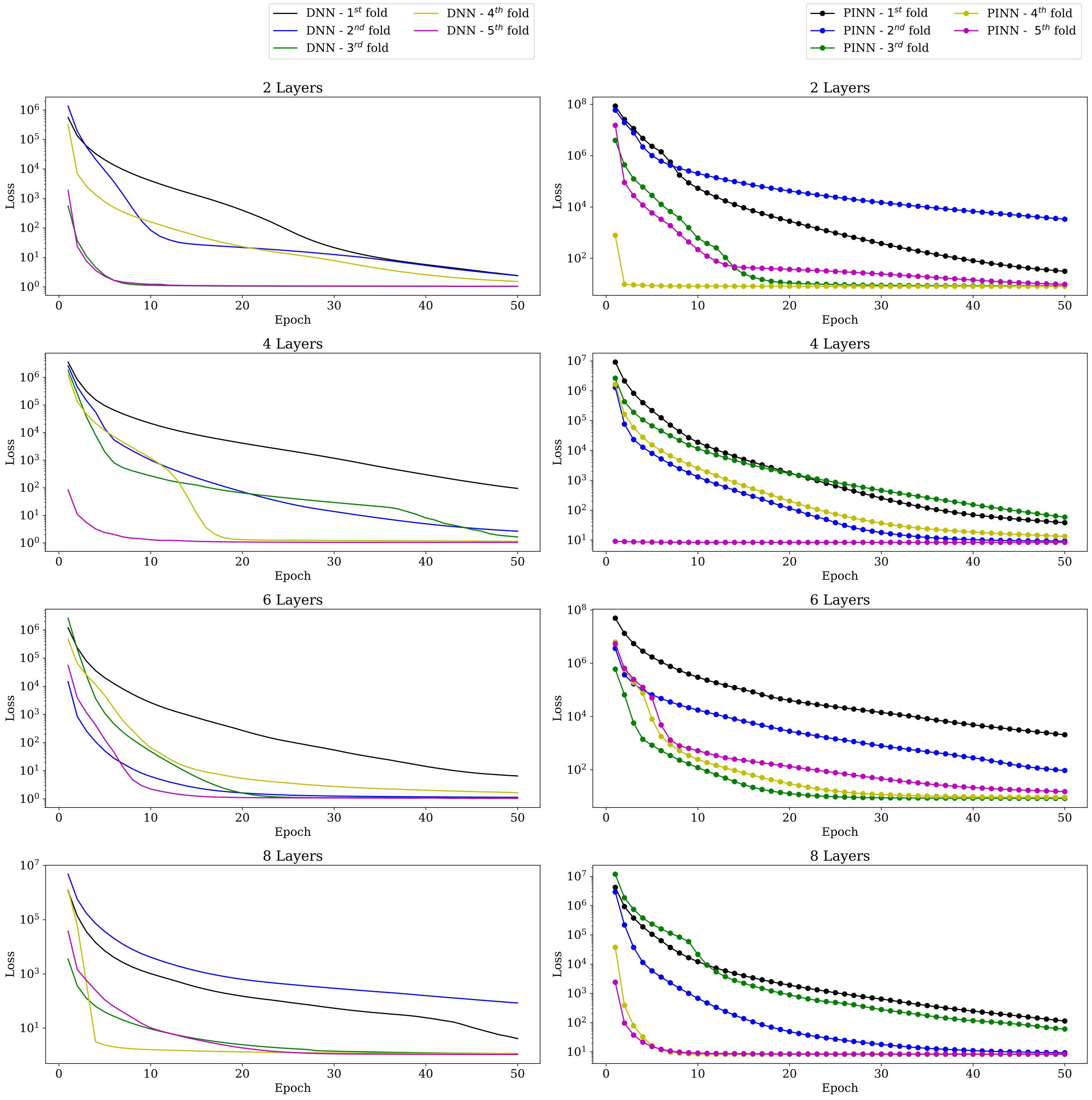

Figure S1. Training loss for DNN and PINN models in 5-Fold Cross-validation using 4000 samples and 50 epochs

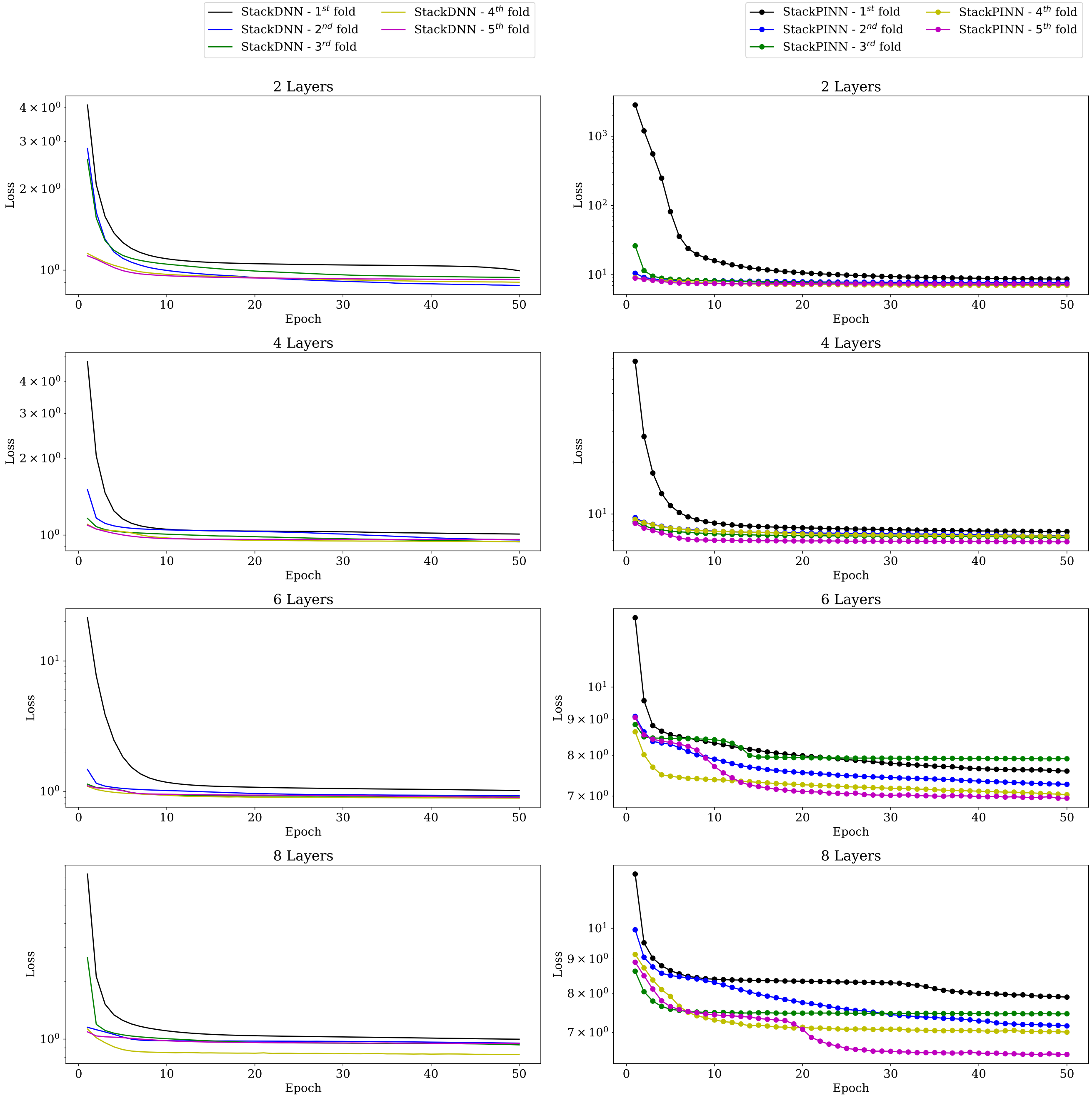

Figure S2. Training loss for StackDNN and StackPINN models in 5-Fold Cross-validation using 4000 samples and 50 epochs

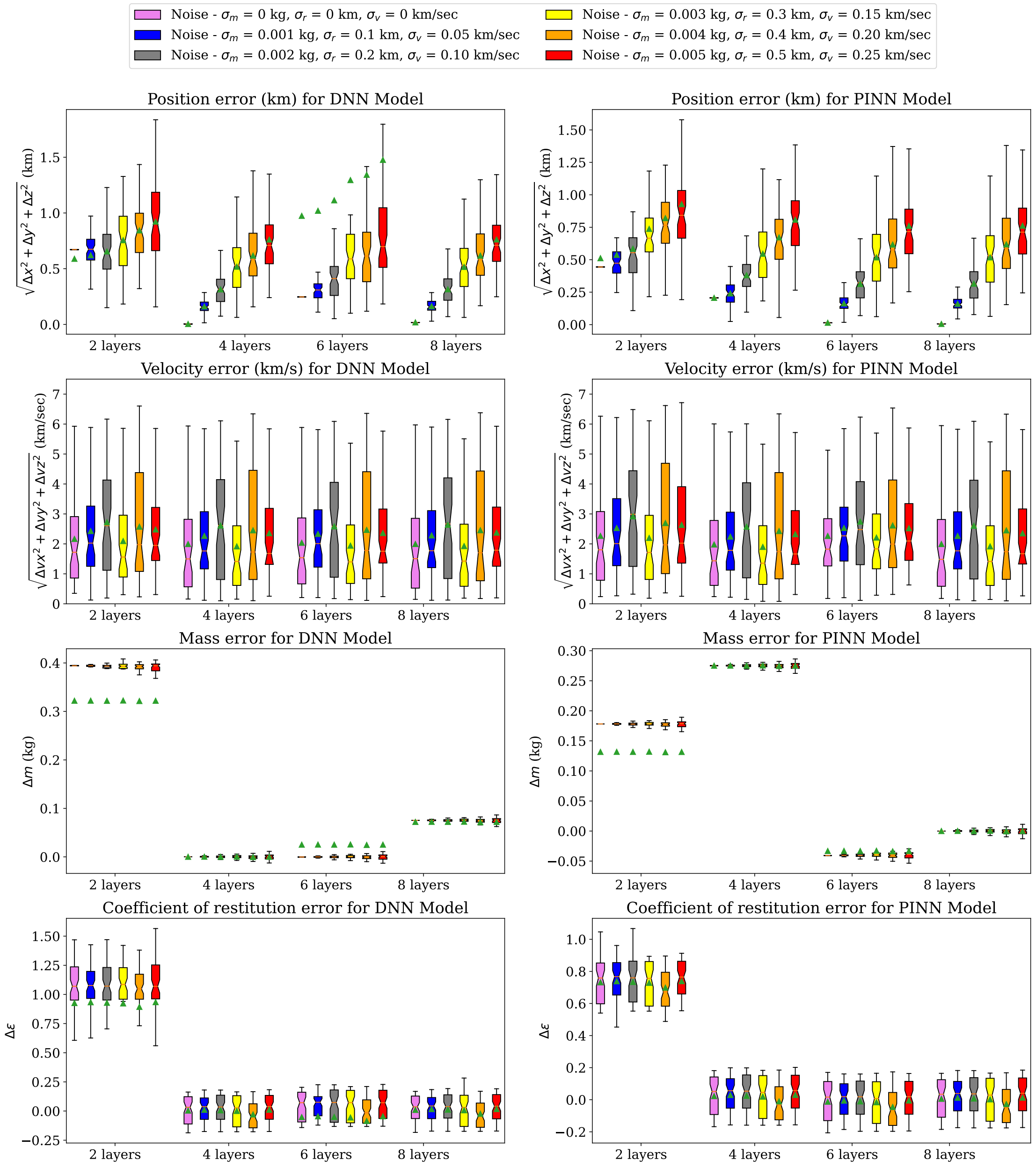

Figure S3. Error comparison of DNN and PINN models trained using 4000 samples and 50 epochs for multiple noise variations in LEMUR Satellites inelastic collision data

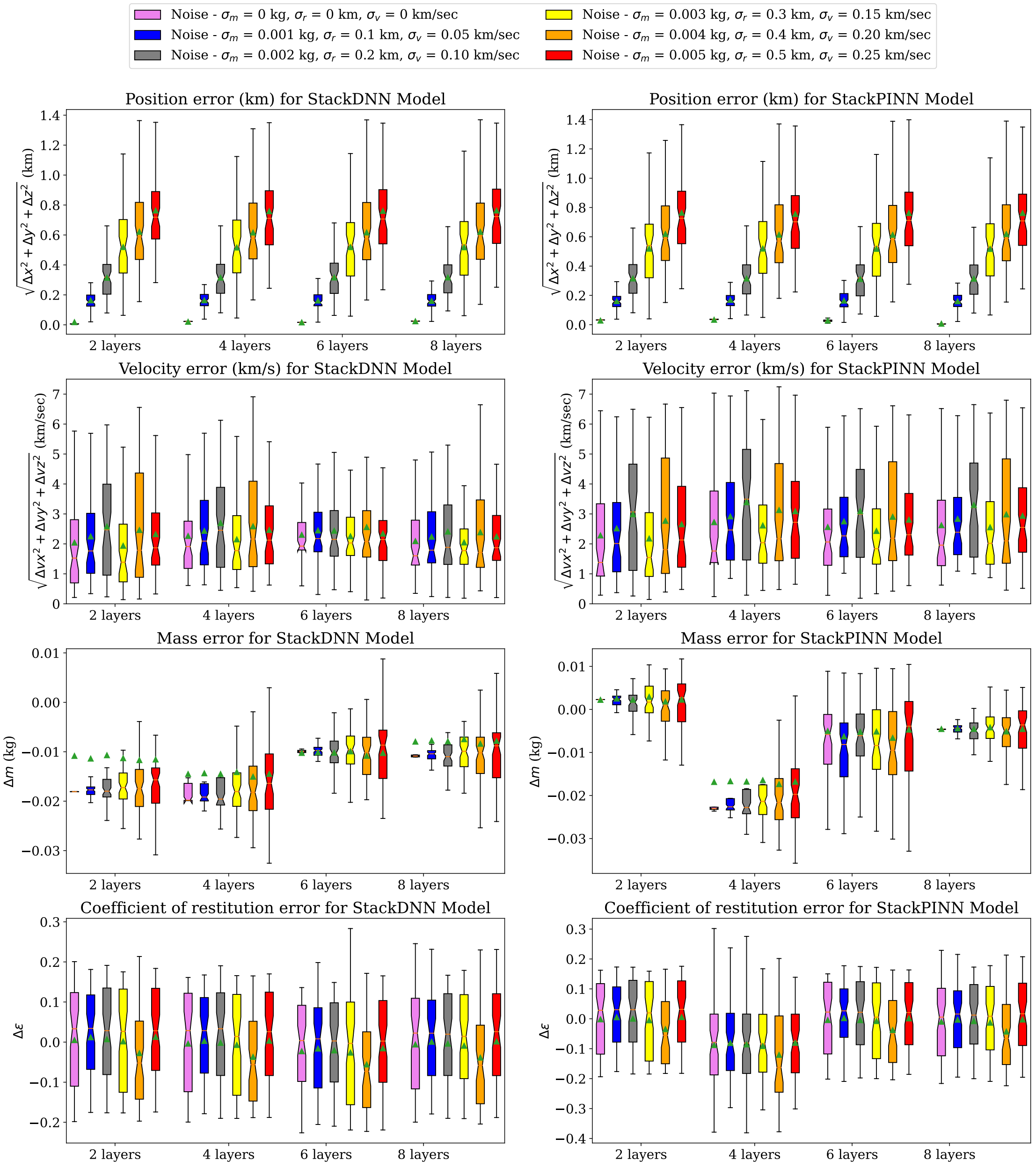

Figure S4. Error comparison of StackDNN and StackPINN models trained using 4000 samples and 50 epochs for multiple noise variations in LEMUR satellites inelastic collision data
